# Impact of intrapartum and postnatal antibiotics on the gut microbiome and emergence of antimicrobial resistance in infants

**DOI:** 10.1038/s41598-019-46964-5

**Published:** 2019-07-23

**Authors:** Terhi Tapiainen, Pirjo Koivusaari, Lauren Brinkac, Hernan A. Lorenzi, Jarmo Salo, Marjo Renko, Hannele Pruikkonen, Tytti Pokka, Weizhong Li, Karen Nelson, Anna Maria Pirttilä, Mysore V. Tejesvi

**Affiliations:** 1 Department of Pediatrics and Adolescence, Oulu University Hospital, 90029 Oulu, Finland; 2 PEDEGO Research Unit and Medical Research Center Oulu, University of Oulu, Oulu, 90014 Finland; 3 Ecology and Genetics, Faculty of Science, University of Oulu, Oulu, 90014 Finland; 4 J. Craig Venter Institute, Rockville, MD 28050 USA; 5 University of Eastern Finland and Kuopio University Hospital, Kuopio, Finland; 6 J. Craig Venter Institute, La Jolla, CA 92037 USA

**Keywords:** Translational research, Microbiome, Metagenomics, Paediatric research

## Abstract

Altogether, 20–30% of women receive intrapartum antibiotic prophylaxis (IAP) to prevent sepsis in infants and 2–5% of newborn infants receive antibiotics due to suspected sepsis. Caesarean section has a long-term impact on the intestinal microbiome but the effects of perinatal antibiotics on gut microbiome in vaginally delivered infants are not well known. We compared the impact of IAP, postnatal antibiotics, or their combination on the gut microbiome and emergence of antimicrobial resistance in a controlled study of 149 newborn infants recruited within 24 hours after birth. We collected 659 fecal samples, including 426 daily samples from infants before discharge from the hospital and 111 follow-up samples at six months. Penicillin was mostly used for IAP and the combination of penicillin and aminoglycoside for postnatal treatment. Postnatal antibiotic groups received *Lactobacillus reuteri* probiotic. Newborn gut colonization differed in both IAP and postnatal antibiotics groups as compared to that in control group. The effect size of IAP was comparable to that caused by postnatal antibiotics. The observed differences were still present at six months and not prevented by lactobacilli consumption. Given the present clinical results, the impact of perinatal antibiotics on the subsequent health of newborn infants should be further evaluated.

## Introduction

*Streptococcus agalactiae*, group B streptococcus (GBS), is an important pathogen causing severe bacterial infections, sepsis and meningitis, in newborn and young infants^1,2^. Maternal vaginal colonization with GBS is the most common route for the acquisition of GBS disease in newborn infants^3^. Altogether 1–2% of newborn infants born to colonized mothers acquire life-threatening sepsis without preventive measures^4^. To prevent GBS disease in newborn infants, in total, 20–30% of women receive clinically effective intrapartum antibiotic prophylaxis (IAP) during vaginal delivery^5,6^. Furthermore, approximately 2–5% of vaginally delivered newborns receive empiric intravenous antimicrobial treatment for suspected neonatal sepsis after birth^7,8^. Universal maternal screening for GBS and active IAP is currently recommended in many countries^9^. Yet, the expert group in the United Kingdom does not recommend active IAP policy since the long-term harm from widespread antibiotic prophylaxis to pregnant women is unknown^10,11^.

Birth is a critical time window for intestinal colonization. Several high-quality studies have shown that Caesarean section (C- section) reduces the diversity of the intestinal microbiome and results in a lower abundance of Bacteroidetes^12,13^. In a recent study comprising patients undergoing appendectomy, the effects of C- section on the intestinal microbiome were still apparent in adulthood^14^. C-section has been associated with immunological changes, such as reduced Th1 chemokine levels^12^, and in epidemiological studies, with increased risk of subsequent asthma^15,16^, obesity^17^, and cardiovascular risk factors^18,19^. In epidemiological studies, both fetal and early-life exposure to antibiotics have been associated with early wheezing or subsequent asthma in children^20,21^. There are limited data on the impact of perinatal antibiotics on the developing gut microbiome in vaginally delivered infants. The clinical efficacy of IAP in preventing GBS sepsis indirectly suggests significant alterations in newborn microbiome^22–24^. The first studies, comprising infants exposed to IAP, have proposed clear effects of IAP on the intestinal microbiome in healthy infants^25–28^.

To produce high-quality evidence regarding the impact of perinatal antibiotic on the developing gut microbiome, we designed a prospective controlled cohort study to compare the impact of IAP, postnatal intravenous antibiotics, or their combination on the gut microbiome and emergence of antimicrobial resistance in vaginally delivered infants. We recruited newborn infants within 24 hours after birth and obtained daily fecal samples during the first week of life until discharge from the hospital, maternal sample before discharge from the hospital, and a follow-up sample from infants at six months.

## Results

### Study population

**Figure 1:**
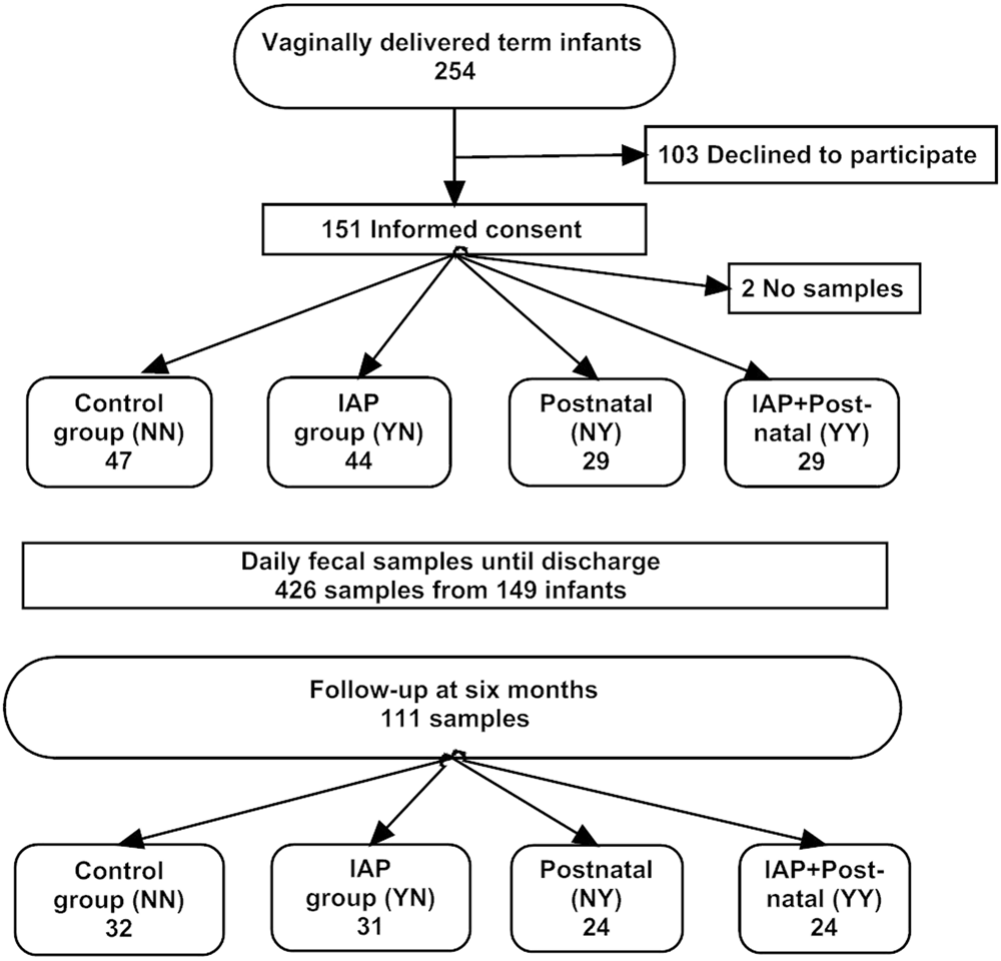
Study design. Antibiotic exposure groups: Control group NN (mother no, infant no), IAP group YN (mother yes, infant no), Postnatal group NY (mother no, infant yes),and IAP + Postnatal group YY (mother yes, infant yes).

We collected 426 daily fecal samples from 149 term infants from the day of birth until discharge and a follow-up fecal sample from 111 infants at the age of six months (Fig. 1). We collected a fecal sample from 122 mothers before discharge. All infants received breastmilk during the first week of life. Infants exposed to postnatal antibiotics received *Lactobacillus reuteri* probiotic until discharge according to local clinical policy. Altogether, 71 infants (48%) were female. In total, 47 newborn infants were not exposed to any perinatal antibiotics (NN group according to the antibiotic exposure: mother no, infant no). Altogether 44 were exposed to IAP in YN group (mother yes, infant no), 29 received intravenous postnatal antibiotics in NY group (mother no, infant yes) and 29 were exposed to both IAP and postnatal antibiotics (YY group). Penicillin was the most frequent antimicrobial agent for IAP (55 of 73, 75%) followed by cefuroxime (12%) and clindamycin (8%) (Table 1). The combination of penicillin and aminoglycoside was the most common choice for postnatal antibiotic treatment (50 of 58, 86%) followed by penicillin alone (14%) (Table 1). Gestational age and birth weight were similar across the antibiotic groups (Table 1). After discharge, 15 infants received another antibiotic treatment before the age of six months, mainly oral amoxicillin or amoxicillin-clavulanate for respiratory tract infections (Table 1).

**Table 1:** Background characteristics of study participants. IAP indicates intrapartum antibiotics prophylaxis. Y = Yes, N = No.

Antibiotics: Mother Y/N Infant Y/N	Control	IAP	Postnatal	IAP + Postnatal	P value^d^
	NN	YN	NY	YY	
	N = 47	N = 44	N = 29	N = 29	
Birth weight (g) mean (SD)	3448 (434)	3589 (517)	3627 (474)	3484 (350)	0.28
Gestational age (d) mean (SD)	279 (8)	279 (8)	283 (7)	279 (11)	0.21
Girls N (%)	23 (49)	15 (34)	16 (55)	17 (59)	0.15
Apgar 10 min N (%)					
4–7	0 (0)	0 (0)	7 (25)	7 (24)	ND^5^
8–10	47 (100)	44 (100)	21 (75)^a^	22 (76)	ND
*L*. *reuteri* during antibiotics	0 (0)	0 (0)	29 (100)	29 (100)	ND
Exclusive breastfeeding (months)	4.6 (1.0)	3.9 (1.3)	5.0 (1.1)	4.3 (1.2)	0.06
Maternal age mean (SD)	29.7 (4.1)	28.5 (5.1)	28.5 (5.2)	29.9 (5.6)	0.47
Maternal weight (kg) mean (SD)^b^	62.6 (10.7)	66.0 (14.9)	62.5 (13.7)	68.4 (12.1)	0.19
Maternal BMI^b^	23.2 (4.2)	24.0 (4.9)	22.9 (4.4)	25.7 (5.0)	0.07
Maternal smoking N (%)	1 (2)	4 (9)	2 (7)	2 (7)	0.56
Maternal chronic medical conditions N (%)					0.15
Total	7 (15)	6 (14)	7 (24)	9 (31)	0.15
Asthma	5 (11)	2 (5)	2 (7)	1 (3)	
Atopic eczema	0 (0)	0 (0)	1 (3)	1 (3)	
Hyperthyreosis	0 (0)	1 (2)	0 (0)	1 (3)	
Hypothyreosis	1 (2)	1 (2)	2 (7)	4 (14)	
IBD	0 (0)	2 (4)	0 (0)	1 (3)	
Epilepsy	1 (2)	0 (0)	0 (0)	0 (0)	
Maternal proton pump inhibitor	0 (0)	0 (0)	2 (7)	0 (0)	0.32
Maternal iron supplementation	13 (28)	10 (23)	22 (76)	18 (62)	0.52
Maternal D-vitamin supplementation	13 (28)	14 (32)	3 (10)	10 (34)	0.14
Maternal diet N (%)					
Vegetarian	2 (4)	1 (2)	0 (0)	1 (3)	0.73
Probiotics	10 (21)	14 (32)	2 (7)	8 (28)	0.07
Antibiotics during pregnancy N (%)	10 (21)	12 (27)	4 (13)	10 (34)	0.92
Intrapartum antibiotics (IAP) N (%)					
Penicillin G		38 (86)		17 (59)	ND
Cefuroxime		4 (9)		5 (17)	ND
Clindamycin		2 (5)		4 (14)	ND
Penicillin + Cefuroxime		0 (0)		3 (10)	ND
No. of IAP antibiotic doses Mean (range)		1.9 (1 to 4)		1.9 (1 to 4)	ND
Postnatal antibiotics started N (%)					
Penicillin G + Tobramycin		25 (86)		25 (86)	ND
Penicillin G			4 (14)	4 (14)	ND
Antibiotics^c^ before 6 mo. N(%)	6 (13)	3 (7)	2 (7)	4 (14)	0.63

^a^One infant had a missing Apgar value at 10 minutes.

^b^In the first trimester of pregnancy, BMI body mass index (normal values 20–25).

^c^Mainly β-lactam antibiotics given during infancy for respiratory infections of the infants, including amoxicillin (N = 6), amoxicillin-clavunate (N = 4), cefuroxime or ceftriaxone (N = 2).

^d^P value indicates the statistical significance of variance analysis for continuous variables, i.e. the difference between all groups, and that of Pearson chi-square test for categorical variables.

ND statistical testing not done as the groups were originally created based on these differences in perinatal antibiotic use. Furthermore, postanatal antibiotics were given only for newborn infants with clinical problems after birth reflected by low Apgar points. *Lactobacillus reuteri* was given only for infants on postnatal antibiotics as a part of their routine clinical care according to the local policy.

### Impact of perinatal antibiotics on the gut microbiome in infants at the phylum level

**Figure 2:**
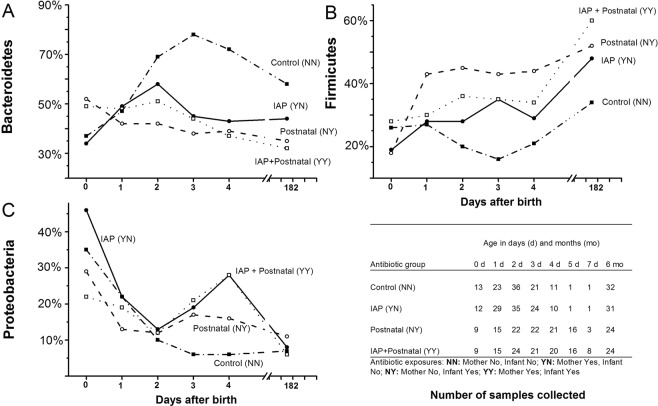
Dynamics of the developing intestinal microbiome of vaginally delivered infants according to perinatal antibiotic exposure during the whole study period. The mean relative abundance of three main bacterial phyla are presented: Bacteroidetes (**A**), Firmicutes (**B**), and Proteobacteria (**C**). The table presents the number of samples and study groups (NN, YN, NY and YY). The statistical comparisons using the data from the whole study period were performed using linear mixed model (P values in the text).

The day-to-day dynamics of the developing intestinal microbiome was different between the groups and remained different until the age of six months (Fig. 2). Using linear mixed model analysis including all study samples, the relative abundance of Bacteroidetes markedly increased in NN group during the first days and remained higher at the age of six months compared to that in the YN, NY and YY groups; *P* = 0.005 between the groups for the whole study period. By the age of three days, the relative abundance of Bacteroidetes phylum in YN, NY and YY groups started to differ statistically from that in the NN group; *P* = 0.04 for the YN group, *P* < 0.001 for the YN group, and *P* = 0.002 for the YY group. Simultaneously, the abundance of Firmicutes decreased in NN group. By the age of three days, the relative abundance of Firmicutes was lower in NN group and differed statistically significantly from YN, YN and YY groups (*P* = 0.048 for YN group, *P* = 0.003 for NYgroup, and *P* = 0.036 for YY group). Using linear mixed model, the difference remained statistically significant at six months between NN group and NY group (*P* = 0.006) and between NN and YY groups (*P* = 0.004). In NN group, the relative abundance of Proteobacteria was the lowest of the four groups, but the difference was not statistically significant, *P* = 0.26 for the whole study period in linear mixed model (Fig. 2).

**Figure 3:**
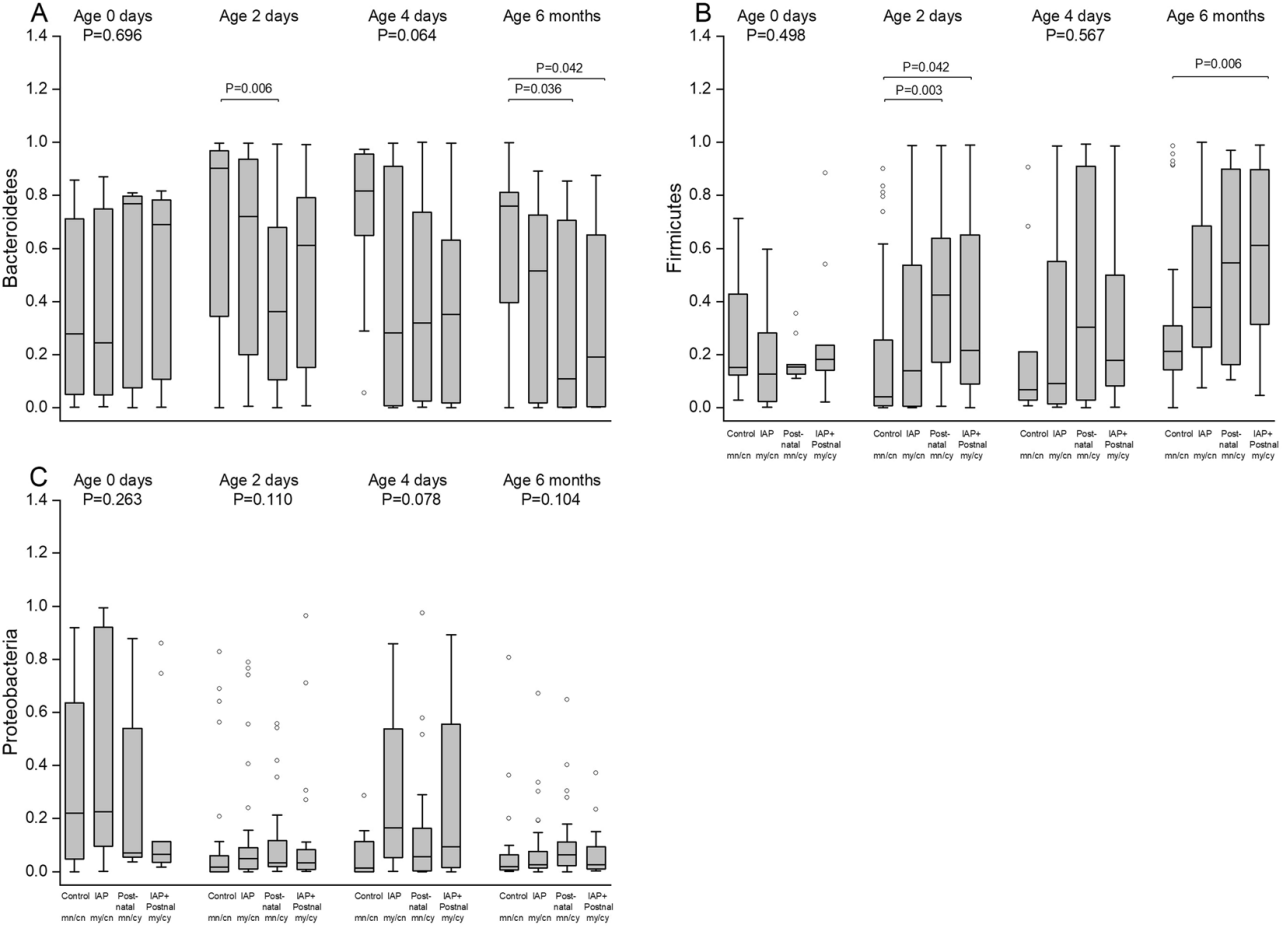
The relative abundances of three main bacterial phyla according to perinatal antibiotic exposure in infants: Bacteroidetes (**A**), Firmicutes (**B**), and Proteobacteria (**C**). The box-plots indicate the median value, interquartile range, and range in control (NN), IAP (YN), in postnatal (NY),and IAP + Postnatal groups (YY). At selected time points, the differences between groups were compared with Kruskal-Wallis test and if P < 0.05 with *post hoc* tests.

In addition to linear mixed model, we used Kruskal-Wallis test with *post hoc* analyses at selected time points (Fig. 3). At the age of two days, the relative abundance of Bacteroidetes differed between NN and NY groups (P = 0.006) and the relative abundance of Firmicutes between NN and NY groups (P = 0.003) and between NN and YY groups (P = 0.042). At six months, the relative abundance of Bacteroidetes differed between NN and NY groups (P = 0.036) and between NN and YY groups (P = 0.042) and the relative abundance of Firmicutes between NN and YY groups (P = 0.006).

### Impact of perinatal antibiotics on the gut microbiome in infants at the genus level

**Figure 4:**
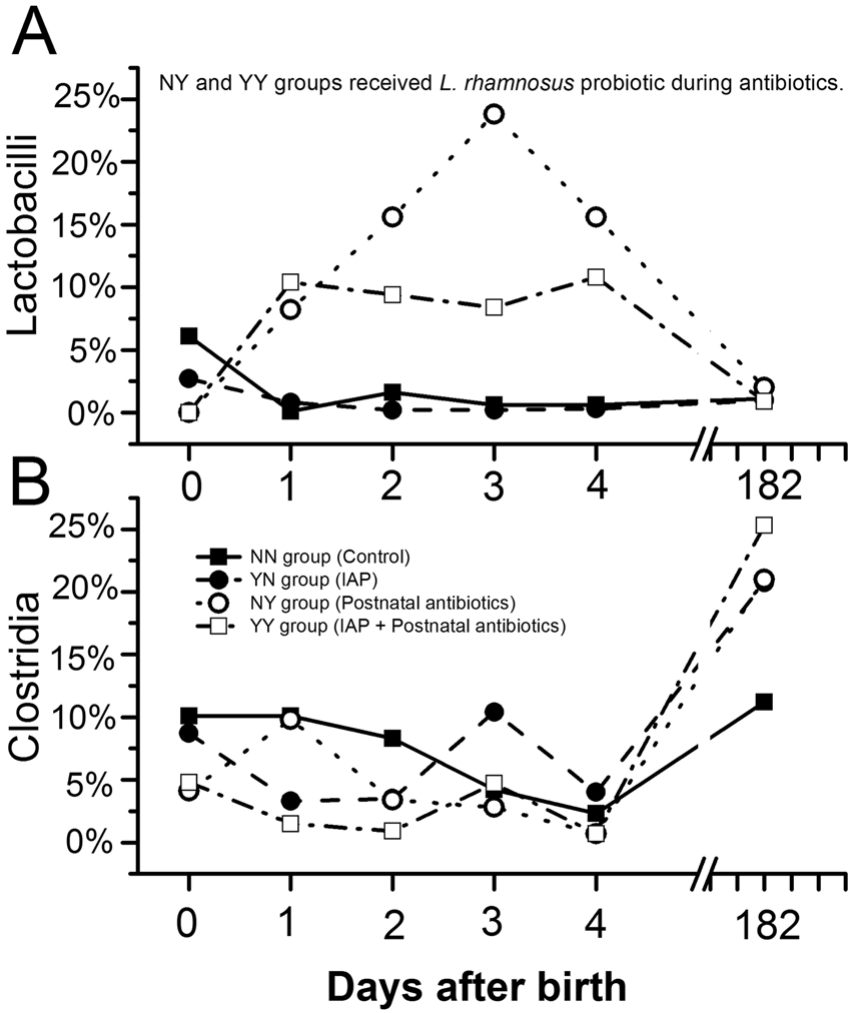
Dynamics of Lactobacillus and Clostridium colonization according to perinatal antibiotic exposure during the whole study period in control (NN), IAP (YN), postnatal (NY),and IAP + Postnatal groups (YY). Infants in NY and YY groups received *Lactobacillus rhamnosus* probiotic during antibiotic treatment according to routine clinical treatment procedure in the hospital. The statistical comparisons using the data from the whole study period were performed using linear mixed model (P values in the text).

**Figure 5:**
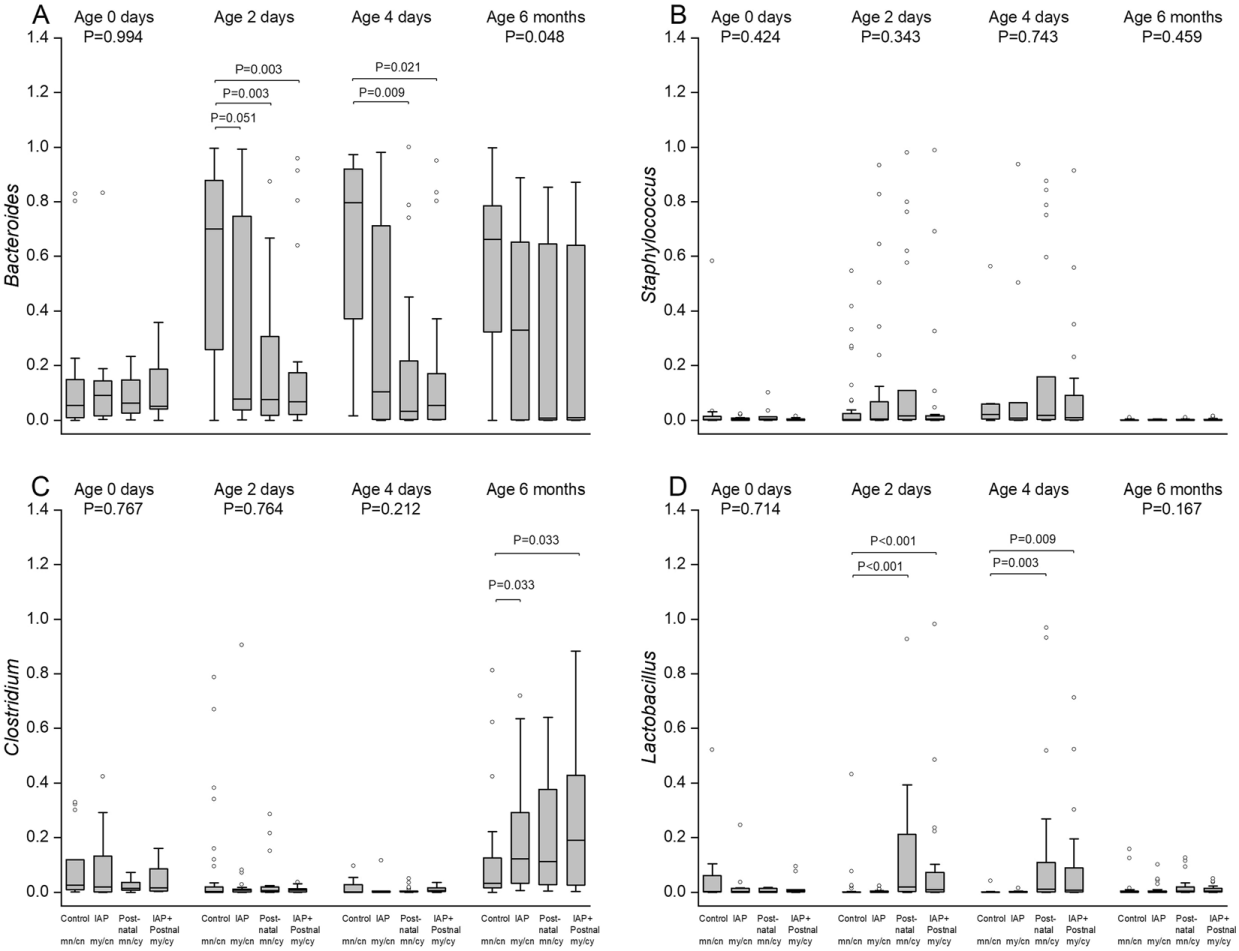
The relative abundances of the bacterial genera mainly explaining the observed phylum level changes after perinatal antibiotic exposure: Bacteroides genus (**A**) for Bacteroidetes phylum, and Staphylococcus (**B**), Lactobacillus (**C**), and Clostridium (**D**) genera for Firmicutes phylum. The box-plots indicate the median value, interquartile range, and range in control group (NN), in IAP group (YN), in postnatal group (NY),and IAP + Postnatal group (YY). At selected time points, the differences between groups were compared with Kruskal-Wallis test and if P < 0.05 with *post hoc* tests.

Only a few bacterial genera accounted for the differences observed at the phylum level. *Bacteroides* was the main genus explaining the increase of Bacteroidetes phylum in NN group, and the *Staphylococcus* and *Clostridium* genera explained the increase of Firmicutes phylum in YN, NY and YY groups (Figs 4 and 5).

Using linear mixed model for comparisons, the relative abundance of lactobacilli peaked in infants receiving postnatal antibiotics and oral probiotic lactobacilli, reaching a relative abundance of 10–20% by day 2 (P = 0.023 between NN and YY group and *P* < 0.001 for NN group and YN group) (Fig. 4). In YN group, unexposed to the *L*. *reuteri* probiotic product, the richness of lactobacilli was similar to that in NN group. By the age of six months, the difference in the abundance of lactobacilli between the groups had vanished. The relative abundance of Clostridia increased over time in all antibiotic groups regardless of consumption of the *L*. *reuteri* probiotic. At the age of six months, the relative abundance of Clostridia was higher in YN group (21%, P = 0.008), in NY group (20%, P = 0.020), and in YYs group (25%, P < 0.001) than that in NN group (11%) (Fig. 4).

In addition to linear mixed model, we used Kruskal-Wallis test with *post hoc* analyses to compare groups at selected time points (Fig. 5). The relative abundance of Bacteroides differed between NN and YN groups (P = 0.003) and between NN and YY groups (P = 0.003) at two days and at four days (Fig. 4). At six months, the relative abundance of Clostridium differed between NN and YN groups (P = 0.033) and NN and YY groups (P = 0.033) (Fig. 5).

### Impact of perinatal antibiotics on alpha and beta diversity

**Figure 6:**
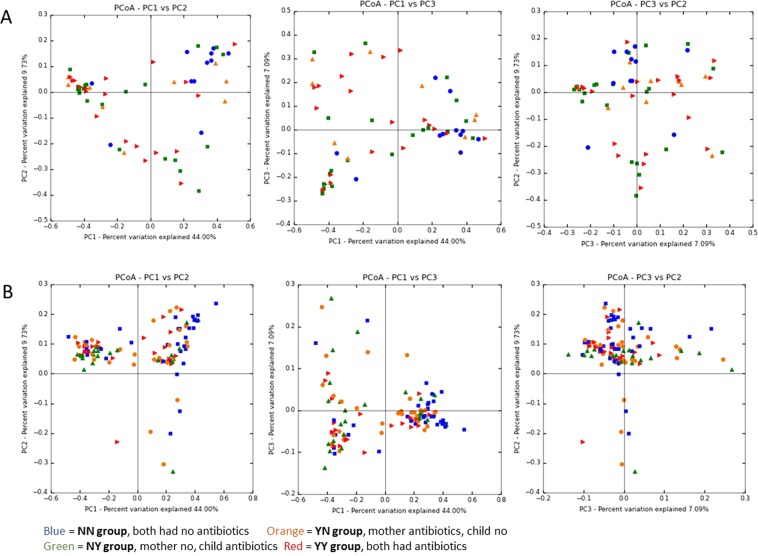
Principal coordinate analysis of fecal microbiota of samples at 96 hours (**A**) and at six months (**B**) according to perinatal antibiotic exposure. Each dot indicates on sample at the selected sampling time. Blue dots indicate NN group (control), orange dots indicate YN group (IAP), green dots NY group (postnatal antibiotics), and red YY group (IAP + Postnatal).

In principal coordinate analysis of microbial composition profiles, the beta diversity profiles of YN, NY, and YY groups started to differ from that of NN group at the age of one day. The fecal microbiomes of the NN samples grouped together both at the age of 96 hours and six months (Fig. 6), whereas the other microbiomes from the YN, NY, and YY did not form any clusters. The daily change of the alpha diversity did not differ between the four different antibiotic exposure groups in a linear mixed model analysis; P = 0.97 for the Chao 1 index. Other measures for microbiome diversity did not differ, either, between the groups during the study period (P = 0.83 for Shannon-Weaver and P = 0.17 for the Simpson index). The mean number of OTUs was 216 (95% CI 161 to 270) in NN group and 260 (95% CI 195 to 325) in YY group on the day of birth, and the development of the number of OTUs did not differ between the groups during the whole study period (*P* = 0.77).

### Antimicrobial resistome of the gut microbiome

For resistome analysis, we included 15 infants from NN group (Control), 10 infants from YY group (IAP + Postnatal), 11 mothers unexposed to perinatal antibiotics and 14 mothers exposed to perinatal antibiotics. The samples were collected 2–4 days after birth. Ten infants in YY group received penicillin (N = 8), cefuroxime (N = 1) or clindamycin (N = 1) as IAP, and penicillin and tobramycin (N = 8) or penicillin (N = 2) as postnatal antibiotics. Six mothers in the perinatally exposed group had received oral antibiotics during pregnancy and four in the unexposed group. Mean number of OTUs in the studied samples was 211 (SD 74) in NN infants, 189 (SD 43) in YY infants, 559 (SD 37) in unexposed mothers and 499 (SD 124) in exposed mothers. Mean Chao1 index was 406 (SD 165) in NN infants, 379 (SD 80) in YY infants, 1401 (SD 125) in unexposed mothers and 1323 (SD 80) in exposed mothers, and Shannon index 5.5 (SD 1.1), 5.2 (SD 0.6), 8.0 (SD 0.24) and 7.6 (0.93), respectively. The mean relative abundance of Bacteroidetes phylum was in 64% (SD 37) in NN group, 27% (SD 35) in YY group, 58% (SD 13) in unexposed mothers and 66%% (SD 20) in exposed mothers.

A total of 241 unique antimicrobial resistance (AMR) genes encoded by 58 bacterial genera, including unclassified, were predicted across all metagenomic samples. Infants exposed to antibiotics showed a significant overall increase in the abundance of AMR genes compared with antibiotic-exposed mothers (P = 1.49 × 10–49) and unexposed babies (P = 5.56 × 10-5). Paired testing, including only samples with available results from the mother-infant pair, showed also a significant overall increase in the abundance of AMR genes compared with antibiotic exposed mothers (p = 6.1 × 10-18) and unexposed infants (p = 1.02 × 10-5).

**Figure 7:**
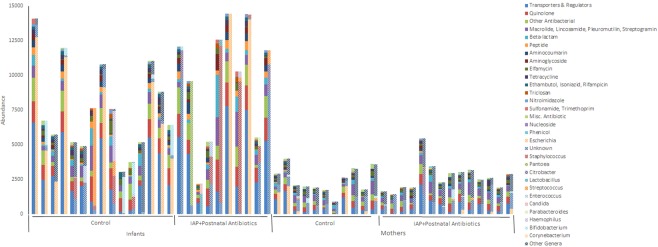
Gut microbiota and antimicrobial resistance (AMR) gene distribution of metagenomic samples. For each sample, the abundance of AMR genes is shown grouped by AMR class (solid stacked bars) next to their corresponding AMR encoding bacterial taxa (patterned stacked bars). Category *Other Genera* include 45 bacterial taxa. The y-axis presents the abundance or normalized counts based on the coverage of sequence reads and scaffold length.

The gut microbiota of infants treated with antibiotics presented significant increases of specific classes of AMR genes, including fusidic acid resistance genes and transporters and regulators, as well as a near significant increase in aminoglycoside-resistance genes. Most AMR genes, overrepresented in antibiotic-treated infants, were encoded by few bacterial taxa that spanned most of the gut microbiota of treated children, including bacteria of the genera *Escherichia* and *Staphylococcus* (29% and 25% of the AMR genes, respectively) (Fig. 7). Only two AMR genes were significantly differentially abundant between the two groups of children after adjusting for multiple testing, most likely due to a low number of samples. Both genes were only present in the Control group, cepA, a beta-lactamase encoding gene, and msrB, a gene encoding for a multidrug efflux pump.

## Discussion

In this prospective controlled cohort study among vaginally delivered infants, the day-to-day dynamics of newborn gut colonization differed in both IAP and postnatal antibiotics groups as compared to that in control group unexposed to antibiotics. The differences in gut microbiome were long-term and not prevented by active probiotic lactobacilli consumption after birth. Furthermore, perinatal antibiotic exposure resulted in the emergence of antimicrobial resistance genes in the intestinal microbiome of infants.

Our prospective study design enabled us to compare the day-to-day impact of IAP, postnatal intravenous antibiotic treatment, or their combination on the developing gut microbiome in a controlled study. Previous, mainly cross-sectional studies have evaluated the effect of sole IAP in groups of 10 to 20 infants. Infants exposed to IAP have been shown to have a lower abundance of Bacteroidetes phylum in the first week of life^25^, lower *Bacteroides* and *Parabacteroides* genera at three months^26^, a higher abundance of Enterobateriaceae at one week^28^, and lower counts of *Bifidobacterium* spp. at the age of one week than control infants unexposed to antibiotics^27^. In the recent high-quality study by Nogacka *et al*.^25^, the impact of IAP on gut colonization was present even by the age of two days, which is similar to our results. Our results further demonstrate that the effect size of IAP, consisting of mostly of narrow-spectrum penicillin given to mothers, was comparable to that caused by several days of wider spectrum intravenous postnatal antibiotic treatment for the newborn infants.

The nature and magnitude of the subsequent health effects of the disturbed gut colonization after perinatal antibiotic exposure are not yet understood^29^. Earlier epidemiological studies have shown that neonatal antibiotic treatment is a risk factor for viral wheezing^21^ and that early oral antibiotic exposure in childhood is linked with subsequent overweight^30^, inflammatory bowel disease^31^, juvenile rheumatoid arthritis^32^, and allergic diseases^33,34^. In an animal model, a single dose of streptomycin decreased the number of intestinal bacteria by 90% and changed 90% of intestinal metabolites^35^. Furthermore, mice exposed to antibiotics before weaning have a disturbed fecal microbiome, and despite the recovery of the intestinal microbiome after stopping antibiotics, the mice had increased fat and total body mass later^36^, and germ-free mice, unexposed to any bacteria, have impaired immune function and higher IgE levels than normal mice^37^. Of note, bacterial metabolites, short-chain fatty acids, are known to influence on the regulatory T cells in the gut^38^.

IAP is effective in reducing early-onset sepsis in newborn infants^5,6^. The present study shows that even narrow-spectrum β-lactam antibiotic, penicillin, mainly used for IAP in our setting, alters the newborn colonization markedly, and the achieved abundance of lactobacilli in infants, receiving probiotic lactobacilli, did not prevent the subsequent long-term changes in gut microbiome. Even though our study does not aim to change the existing clinical guidelines for IAP, the present results do support the ongoing efforts to find a more specific alternative for early-onset sepsis prevention, such as maternal group B streptococcal immunization^39^, to avoid the potential harmful effects of perinatal antibiotics on the intestinal microbiome and subsequent health.

In addition to the observed changes in the intestinal microbiome, we observed a significant increase of antimicrobial resistance genes in infants exposed to perinatal antibiotics. The numerous AMR genes found in the early intestinal microbiome after exposure to IAP and postnatal antibiotics in our study is analogous to that reported by Nogacka *et al*. in infants exposed to IAP^25^. In our study, AMR genes were more abundant in the gut microbiome of the newborn infants exposed to perinatal antibiotics than in the exposed mothers. Thus, our results suggest that newborn infants may be at a particular risk for the selection of resistant strains after antibiotic exposure, possibly due to a limited number of bacterial species present in the newborn gut, allowing a rapid selection and a fast relative increase of few bacterial strains with suitable AMR genes after antibiotic exposure.

The strength of our study is its prospective clinical study design that enabled us to show the day-to-day dynamics of gut colonization with a follow-up at six months. Furthermore, we were able to compare the impact of sole IAP to that of postnatal intravenous antibiotics, as we had three different prospectively collected antimicrobial exposure groups, and a control group, for comparisons. The next-generation sequencing of the bacterial 16S rRNA gene is becoming a routine method in clinical research, and a large number of samples, can be analyzed in a feasible manner, which enables meaningful statistical analysis. Finally, we performed metagenome sequencing to demonstrate the impact of perinatal antibiotics on the emergence of AMR genes in infants. The limitation of the present study is that we present the relative changes in microbial communities. There could be, however, significant clinical effects that are dependent on the absolute number of bacterial counts. Only newborn infants with clinical problems received postnatal antibiotics, which may interfere with the results. Our sample size did not allow us to evaluate the association of a specific gut microbiome alteration with a particular clinical outcome in this unselected cohort of newborn infants.

In summary, we show that narrow-spectrum β-lactam, given to women during vaginal delivery to prevent newborn sepsis, has a rapid, marked, and long-term impact on the developing gut microbiome in infants. The effect size was similar to that observed after postnatal intravenous antibiotics. In previous animal models, the disturbed early gut microbiome has been linked with allergic diseases, obesity, and poor gut health. Thus, given the present clinical results, we propose that the impact of IAP on long-term health needs to be evaluated in the future.

## Methods

### Study design and study population

This was a prospective controlled cohort study among term, vaginally delivered infants followed from birth until the age of six months. We recruited the study participants in the Department of Women and Children in Oulu University Hospital, Oulu, Finland from February 2014 to June 2015. Oulu University Hospital serves as the only delivery hospital in the area, with approximately 4,000 births annually. The Regional Ethics Committee of the Northern Ostrobothnia Hospital District, Oulu University Hospital, Oulu, Finland, reviewed and approved the study plan. All experiments were performed according with relevant regulations and guidelines. We enrolled only newborn infants whose parents gave written informed consent.

### Antibiotic exposures and samples

We collected fecal samples from infants in four different groups depending on their perinatal antibiotic exposure: Control group (NN group), IAP group (YN group), Postnatal antibiotics group (NY group), and IAP + Postnatal antibiotics group (YY group). NN group was unexposed to maternal antibiotics within one week before delivery, IAP during delivery, and postnatal antibiotics during the first week of life. YN group was exposed to sole intrapartum antibiotic prophylaxis. NY group received postnatal intravenous antibiotics starting within 24 hours after birth. YY group was exposed to both IAP and postnatal intravenous antibiotics. *Lactobacillus reuteri* probiotic product, using a daily dose of 10^8^ CFU, was administered to all infants receiving postnatal antibiotics until discharge. All antibiotics and probiotics were chosen and given as clinically indicated by the attending physicians according to local policy. Midwives and neonatal nurses collected daily fecal samples from diapers until discharge. Mothers provided a fecal sample for analysis after birth or soon after discharge. Samples were stored at − 20 °C until microbiome analysis.

### Next-generation sequencing of bacterial 16S rRNA gene and bioinformatics

We used the QIAamp Fast DNA Stool Mini Kit (Qiagen, USA) according to the manufacturer’s protocol to extract DNA from the fecal samples of the infants and their mothers. The samples were stored the DNA at −20 °C until used for next generation sequencing of bacterial 16S rRNA gene. DNA was quantified using a Nanodrop spectrophotometer. We chose to use primers F519 (5-CAGCMGCCGCG GTAATWC-3) and R926 (5-CCGTCAATTCCTTTRAGTTT-3) to amplify the V4-V5 region of the 16S small-subunit ribosomal gene (16S rRNA). The F519 primer contained an Ion Torrent pyrosequencing adapter sequence A (Lifescience Technologies, USA), a 9-bp unique barcode sequence, and one nucleotide linker, while the R926 primer contained an Ion Torrent adapter trP1 sequence. We have earlier used similar methodology for NGS and bioinformatics of fecal samples obtained in infancy^40^.

We performed triplicate PCR reactions, each containing 1x Phusion GC buffer, 0.4 µM of the forward and reverse primers, 200 µM dNTPs, 0.5 U of the Phusion enzyme (Finnzymes. Finland), and 10 ng of genomic community DNA as the template together with molecular-grade water in a total reaction volume of 25 µl. We used the following cycling conditions: 30 cycles at 98 °C for 10 s, 64 °C for 30 s, and 72 °C for 20 s after an initial denaturation at 98 °C for 3 min. The triplicate pooled PCR amplified reactions were purified using the AMPure XP PCR clean-up kit (Agencourt Bioscience, CA, USA). We measured DNA concentration with a Bioanalyzer DNA chip (Agilent Technologies, CA, USA). Individual samples were pooled in an equivalent amount, size selected with the BluePippin automated electrophoresis system (Sage Science, MA, USA) on 1.5% agarose gel, and purified twice with an AMPure XP kit. The achieved DNA concentrations were measured with a Bioanalyzer DNA chip and sequenced on a 316 v2 chip using Ion Torrent 400 bp chemistry (Life Technologies, USA) using to 15 pM sample concentration.

To characterize the microbiomes of the fecal samples, we sequenced the hypervariable regions V4-V5 of the 16S rRNA gene using Ion Torrent. We then analyzed the Ion Torrent sequences with QIIME1.9. Taxonomy was given using gut-specific microbiome HITdb^41^. We used Shannon-Weaver, Simpson, and Chao1 indices to estimate the alpha diversity of the microbiome. We have submitted the Ion Torrent sequences to Genbank with an accession number SRP152384.

We analyzed the Ion Torrent sequences with QIIME. The sequences were binned according to sample-specific barcodes using the QIIME split_libraries.py tool. Then the barcode and primer sequences were trimmed and filtered for quality using the default parameters. We removed chimeric sequences with the Usearch quality-filtering tool in QIIME using the rRNA16S.gold.fasta reference database. We clustered the sequences into operational taxonomic units (OTUs) with a similarity threshold of 97% in an RDP Naive Bayesian Classifier. The OTUs represent taxonomic units based on differences between sequences of bacterial DNA data. The total number of OTUs is an estimate of the total number of bacterial species in the sample, but is not the same concept as the number of different microbiological species. The phylogenetic trees were created from NAST-trimmed aligned sequences in FastTree2. We used the Biom formatted table in QIIME to construct OTU table. All the samples were rarefied to 1,379 sequences prior to the OTU-based analysis because it was the lowest number of readings observed in the community.

### Metagenome sequencing and bioinformatics

Altogether, 54 total fecal samples were processed for metagenomic taxonomic classification and antimicrobial resistance profiling. In total, 25 samples (14 infant-mother pairs) were from the NN group, and 29 samples (15 infant-mother pairs) were from the YY group. Infant samples corresponded to the last fecal sample obtained prior to discharge (days 3–4 for controls and days 5–7 for the treated group). For metagenome sequencing, DNA was extracted from fecal samples using the MOBio Powersoil DNA Extraction kit (MOBio Labs, Carlsbad, CA, USA). Paired-end bar-coded libraries were prepared from extracted DNA using the NexteraXT kit (Illumina) and sequenced on a NextSeq instrument (Illumina), generating approximately 1 Gb of shotgun sequence data (2 × 150 bp paired-end reads) per sample. Three maternal samples from the control and one infant sample from the treated group did not generate enough sequence data and were discarded.

Metagenomic raw reads were filtered by removing low-quality bases and adapter sequences using Trimmomatic^42^. High-quality paired-end reads longer than 100 bp were then mapped to the human reference genome (hg38) with BWA-MEM^43^ and removed if they mapped concordantly with an alignment score of at least 60. Remaining reads were mapped to a reference genome database, compiled and curated from the NCBI RefSeq genome database, consisting of genomes from viruses, bacteria, archaea, fungi, and other small microbial organisms. Mapping was performed with BWA-MEM. Top scored (≥75), concordantly mapped alignments were used for taxonomy profiling, which calculated the relative species abundance at different taxonomic ranks using a voting scheme. For functional analysis, high-quality reads were assembled into scaffolds using metaSPAdes^44,45^ (Parameters:–meta–only-assembler). Scaffolds shorter than 250 bp were removed. Protein-coding genes were predicted using Prodigal^46^ and then processed using the Resistance Gene Identifier (RGI)^47^ in strict mode to predict the antibiotic resistome of each metagenomic sample using the CARD^47–49^. To estimate the number of AMR gene copies present in a sample, the total amount of sequencing data mapped to every scaffold was divided by the corresponding scaffold length. The resulting normalized coverage per scaffold was used as an estimated copy number for every gene encoded in the scaffolds. For the comparative analysis of AMR gene abundance across subjects, estimated gene counts per sample were further normalized by the total number of reads generated for each sample. Statistical analysis of the overrepresentation of AMR gene categories between either antibiotic-treated or control groups was performed with a two-tail t-test. The identification of individual AMR genes differentially represented between treated and control groups of infants was carried out with the R package EdgeR^50^. R package VEGAN^51^ was used for nonmetric multidimensional scaling (NMDS) analysis (metaMDS), the calculation of the Bray-Curtis distance matrix (vegdist), and the permutational multivariate analysis of variance (PERMANOVA) test (adonis2).

### Statistical analysis

We analyzed the influence of early exposure to antibiotics on the characteristics of the intestinal microbiome, including the number of operational taxonomic units (OTUs), the indices of alpha diversity, and the relative abundances of the main phyla and genera during the first week of life and at six months. We used a linear mixed model since it is the recommended statistical analysis for repeated measurements over time in clinical series, often complicated by missing data in certain time points^52^ We used a linear mixed model analysis with random intercept and first-order autoregressive covariance structure for repeated measurements (sample age in days) to model the microbiome characteristics, as dependent variables between the antibiotic treatment groups (Control, IAP, Postnatal, and IAP + Postnatal). The random intercept and repeated measurements were nested within subjects. The group-by-day interaction was used to test differences between antibiotic groups at each sample age point. In addition to linear mixed model analysis, we used Kruskal-Wallis test to compare the differences at selected time points with Tukey post hoc tect when P < 0.05. The differences in background characteristics between groups were compared using variance analysis for continuous variables and chi-square tests for categorical variables. We performed statistical analyses of bacterial 16S rRNA bacterial gene data with IBM SPSS Statistics 25 for Windows (Armonk, NY, USA, IBM Corp.). Statistical analysis of the overrepresentation of AMR gene categories between either the antibiotic-treated or control groups was performed with a two-tail t-test. The identification of individual AMR genes differentially represented between treated and control groups of infants was carried out with the R package EdgeR.

### Public databases

We have submitted the Ion Torrent sequences to Genbank with an accession number SRP152384. Metagenomic data were submitted to NCBI SRA with an study accession number SRP198974 and run Accession Number: SRR9095167 - SRR9095214. Data will be released on publication.
